# The DID-guide: A guide to developing digital mental health interventions

**DOI:** 10.1016/j.invent.2024.100794

**Published:** 2024-12-07

**Authors:** E.C.A. Mertens, J.-L. Van Gelder

**Affiliations:** a Department of Criminology, Max Planck Institute for the Study of Crime, Security and Law, Günterstalstrasse 73, 79100 Freiburg im Breisgau, Germany; b Netherlands Institute for the Study of Crime and Law Enforcement, De Boelelaan 1077, 1081HV Amsterdam, the Netherlands; c Institute of Education and Child Studies, Leiden University, Wassenaarseweg 52, 2333AK Leiden, the Netherlands

**Keywords:** e-Mental health, Digital mental health interventions, Smartphone applications (apps), Immersive Virtual Reality (VR), Theory translation, Development guidelines

## Abstract

The opportunities technology offers for improving mental health have led to a surge in digital interventions. A pivotal step in the development of such interventions involves translating theoretical intervention techniques into specific technological features. However, practical guidelines on how to approach this translation are currently underdeveloped. To support efforts to develop digital mental health interventions, from theoretical inception to an actual digital intervention, we present the Digital Intervention Development Guide (DID-Guide). The DID-Guide is structured into two distinct phases. Phase 1 establishes the intervention's theoretical foundation, outlining the steps for developing a theoretical intervention framework. Phase 2 translates this theoretical framework into actionable technological features, that make up the intervention. We break down the DID-Guide's two phases into a series of actionable steps, accompanied by concrete examples from a recent intervention that can be delivered through both a smartphone app and Virtual Reality. The DID-Guide serves as a comprehensive resource for creating impactful digital mental health interventions, while also facilitating collaboration and communication among a diverse range of stakeholders, including researchers, clinicians, and software developers.

## Highlights

• The DID-Guide can support the development of digital mental health interventions.
• Conceptualization of the intervention is an important step in development.
• The fit between intervention content and technology's characteristics is crucial.

## 1 Introduction

Digital Mental Health (DMH) interventions have proliferated in recent years. Technologies, such as smartphone applications (apps), Virtual Reality (VR), computer-interfaces, and wearables (e.g., smartwatches), offer unique opportunities that often cannot be achieved in traditional face-to-face settings. Such technologies can, for example, provide remote intervention, automate the delivery process, and generate realistic simulated immersive scenarios. Developing digital interventions is challenging, however, as it requires the translation of, generally abstract, theoretical concepts into concrete technological features (Rabbi et al., 2016) and different delivery technologies may also require different implementation strategies.

Guidelines for developing interventions and their features have struggled to keep pace with recent technological advancements. To address this gap, we developed the Digital Intervention Development Guide (DID-Guide). This guide provides a step-by-step approach for anyone who wants to develop a DMH intervention, e.g., researchers, clinicians, and software developers. The DID-Guide offers a dual-purpose resource, enabling the development of both the theoretical foundation and the technological features of a digital intervention, while also providing a shared framework for communication among stakeholders.

### 1.1 Intervention development frameworks

**Fig. 1:**
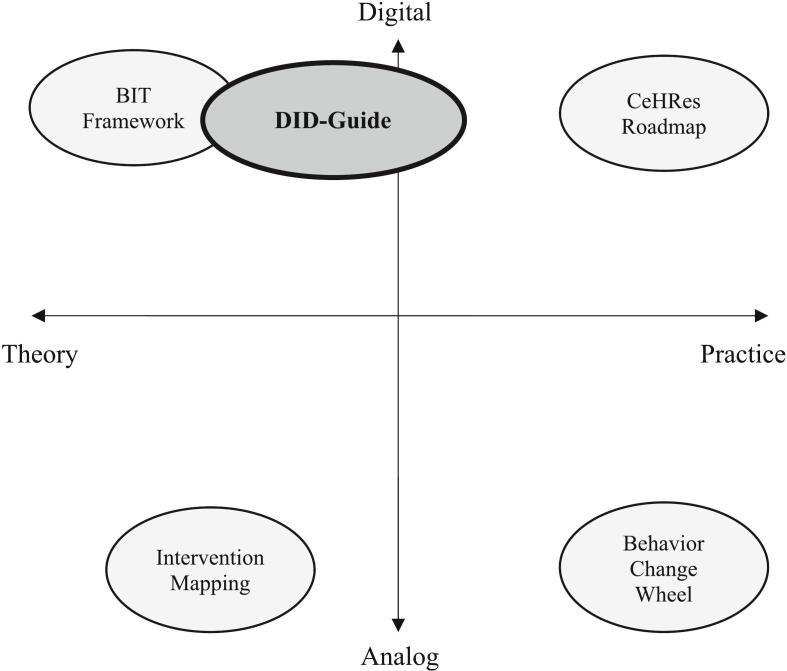
A Visual Comparison of Intervention Development Frameworks on the Dimensions of *Process*, from Theory-Focused to Practice-Focused (X-Axis), and the Level of *Digitalization*, from Analog to Digital Interventions (Y-Axis).

Multiple frameworks that guide the development of mental health interventions exist. These frameworks can be distinguished along two different dimensions: 1) the degree to which the development *process* is guided by theory (i.e., theory and empirical evidence) versus practice (i.e., involving stakeholders and the intervention context), and 2) the degree to which they focus on *digitalization*, that is, analog versus digital interventions. To illustrate, we classify and describe four established intervention development frameworks according to this distinction (see also Fig. 1).

The Center of eHealth Research (CeHRes) roadmap (Van Gemert-Pijnen et al., 2011; Kip et al., 2024) emphasizes a practice-driven development process and focuses on digital interventions. It advocates a holistic approach, in which the stakeholders and context are closely involved in the process. The roadmap aims to guide the planning, coordination, and participatory design process of digital interventions. As such, the CeHRes roadmap can be classified as a practice-driven framework for digital interventions.

The Behavior Change Wheel (Michie et al., 2011) also devotes specific attention to a practice-driven development process, as it stresses collaborations with stakeholders and consideration of an intervention's context. However, it does not address digitalization of an intervention. The framework provides a basis for a systematic analysis for intervention selection by underscoring the importance of taking the context in which the behavior occurs as well as in which the intervention will be implemented into account. Hence, the Behavior Change Wheel can be classified as a practice-driven framework for analog interventions.

Intervention Mapping (Kok et al., 2017) uses a more theory-driven development process and does not discuss digitalization either. While there is attention for the involvement of stakeholders and context, the framework underlines the use of theory and empirical evidence to identify determinants of targeted behaviors, and to select relevant behavior change techniques. Therefore, we classify it as a theory-driven framework for analog interventions.

The Behavioral Intervention Technology framework (BIT framework; Mohr et al., 2014), finally, also uses a theory-driven development process and completely focuses on digital interventions. The framework consists of two distinct components. The first component focuses on defining the intervention goals and identifying the behavioral change techniques required to achieve them. The second component involves translating these techniques into tangible technological features that can be implemented to drive behavior change. Thus, the BIT framework can be classified as a theory-driven framework for digital interventions.

The DID-Guide combines a theory-driven development process with elements of practice-driven processes and concentrates on digital interventions. It applies a theory-driven development process for building the intervention's theoretical foundation and employs elements of a practice-driven process when converting this theoretical foundation into technological features by explicitly addressing involvement of stakeholders, such as software developers, and consideration of the intervention's context. Accordingly, the DID-Guide can be classified as a framework in between theory- and practice-driven processes for the development of digital interventions.

### 1.2 The Digital Intervention Development Guide (DID-Guide)

**Fig. 2:**
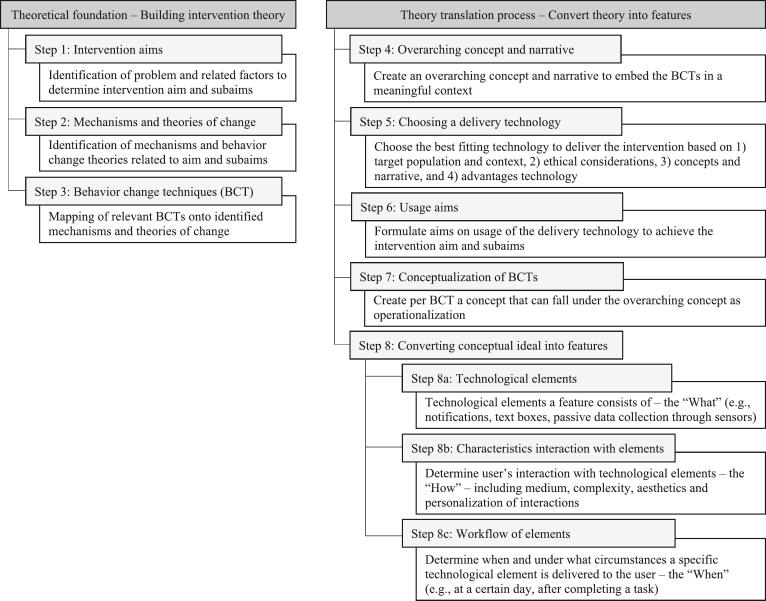
Overview of the DID-Guide Showing the Theory Foundation Phase and the Theory Translation Phase Including the Steps of Each Phase.

The DID-Guide aims to support the development of DMH interventions for anyone who intends to develop such an intervention and provides a common framework for communication among stakeholders. Given the importance of translating theory into effective technological features (Rabbi et al., 2016), it places strong emphasis on the process of converting intervention theory into concrete technological features – the fundamental components of a digital intervention. It builds on a solid base of existing frameworks for intervention development, in particular the four discussed above, and describes two phases of DMH intervention development (see Fig. 2). The first phase establishes the intervention's theoretical foundation. It outlines three steps for developing a theoretical framework, including the intervention aims. The second phase translates this theoretical framework into actionable technological features, that make up the intervention. It consists of eight steps that guide intervention developers through the complete translation process – from conceptualization and choosing a delivery technology to intended use of the technology and step-by-step development of technological features.

#### 1.2.1 How to use the DID-Guide

The two-phase structure of the DID-Guide facilitates a flexible use. When developing a DMH intervention, one can follow all steps of the guide to build a digital intervention from ideas to a working product. Alternatively, in case the theoretical foundation of an intervention is already developed, users can go straight to the second phase of the guide. For example, there is a trend to convert in-person interventions into blended or digital versions. In these cases, the theoretical foundation is already present, and thus only the second phase of the DID-Guide is relevant.

In the next section, we elaborate on the different steps of the DID-Guide. Definitions of concepts used in the guide are listed in Box 1. To illustrate each step, we present examples from a recently developed smartphone- and VR-based mental health intervention: FutureU.

Box 1 **Concepts, abbreviations, and definitions in the DID-Guide.**

**Table:** 

Concept	Definition
Digital Mental Health intervention (DMH Intervention)	Mental health intervention that uses one or more types of technology for its delivery.
Behavioral Change Technique (BCT)	An observable and replicable intervention technique designed to change behavior, i.e., an ‘active ingredient’ of an intervention.
Overarching concept	An idea that can integrate the different components of the intervention's theoretical foundation.
Narrative	A storyline with characters and/or events that creates a context and allows the technology to ‘tell a story’.
Features	The building blocks of a DMH intervention that consist of: *Technological elements* (e.g., text boxes) with which users can *interact* (e.g., type a response) and are offered in a predefined order, i.e., a *workflow* (e.g., first present a question, then present the option to type text in a text box).
Technological Elements	The building blocks of a feature. In other words, the technological aspects that need to be presented (e.g., text box) or programmed (e.g., tracking steps) in order to build a specific feature.
Interactions	The way technological elements are presented to users and how users can engage with them
Workflow	When and under what circumstances a technological element (or feature) is delivered.
User Experience (UX)	The design of interactions with a feature (and the complete digital intervention) that are functional and logical for the user.
User Interface (UI)	The look, appeal, and aesthetics of a feature (and complete digital intervention).

Alt-text: Box 1

## 2 DID-Guide: building an intervention's theoretical foundation

The first phase of the DID-Guide constitutes the theoretical foundation of the intervention and consists of three steps that are based on the theory-building steps in other frameworks (e.g., Behavior Change Wheel in Michie et al., 2011; Intervention Mapping in Kok et al., 2017).

### 2.1 Step 1: intervention aims

The development of an intervention starts with a problem in a target population or socio-ecological context that needs to be addressed. A problem can be identified, for instance, through research indicating that certain behaviors lead to disadvantageous outcomes or by asking people which problems or challenges they encounter in (specific domains of) their lives (i.e., needs assessment).

Insight into how to address the identified problem can be gained by using different strategies, such as searching the literature for information on etiology, mediating mechanisms, and/or risk and protective factors (i.e., determinants of behavior), and/or by engaging with the target population and other stakeholders. The problem, its behavioral and environmental causes, and the determinants of these causes can be depicted together in a *logic model of the problem* (Kok et al., 2017). Based on this model, an overarching intervention aim and potential supporting sub-aims are formulated (see Case Example Step 1).

### 2.2 Step 2: mechanisms and theories of change

After determining the aims of the intervention, knowledge on the factors that affect and maintain the targeted behavior(s) should be acquired in order to identify potential change methods for the intervention (Kok et al., 2017). To this end, behavior maintaining and change theories and mechanisms for each aim need to be identified, culminating in a *logic model of change* (Kok et al., 2017). This too can be achieved through strategies such as searching the literature (e.g., Direito et al., 2018) and/or by consulting relevant populations and stakeholders (e.g., Poot et al., 2023) (see Case Example Step 2).

### 2.3 Step 3: Behavior Change Techniques (BCTs)

Once mechanisms and theories of change have been identified, intervention techniques that have the potential to change behavior, i.e., Behavior Change Techniques (BCTs), can be mapped onto them. These BCTs, such as exposure, goal setting, and monitoring of behavior, can be considered the ‘active ingredients’ of an intervention that (are intended to) bring about behavioral change (Michie et al., 2013). The process of identifying and mapping relevant BCTs can be supported by a BCT taxonomy – an extensive list of hierarchically structured intervention techniques (e.g., Michie et al., 2013; updated by Marques et al., 2023) – and by studies examining effective intervention components (e.g., Fanning et al., 2017) (see Case Example Step 3).

**Table:** 

**FutureU Case Example: Steps 1, 2, and 3.**
FutureU is an intervention initially developed to target delinquency among convicted male offenders (e.g., Van Gelder et al., 2022), although it also shows promise for other populations (e.g., Mertens et al., 2024).

**Step 1: Intervention aims.**
Delinquency carries high social and financial costs. A key driver of delinquency is the tendency to prioritize the present and to ignore or neglect the longer term consequences of one's behavior (Gottfredson & Hirschi, 1990; Van Gelder & Frankenhuis, 2025). This tendency may be related to the degree to which people identify with their ‘future self’ (e.g., Hershfield, 2011; Van Gelder et al., 2015, Van Gelder et al., 2022). Hence, future self-identification is a possible mediating mechanism underlying delinquent behavior. Based on this assumption, we defined “decreasing delinquency” as the overarching intervention aim, and 1) stimulating future-oriented thinking and behavior, and 2) strengthening future self-identification, as subaims.

**Step 2: Mechanisms and Theories of Change.**
We identified several relevant mechanisms and theories of change to achieve these aims. For instance, for the subaim “strengthen future self-identification” research suggests that ‘vividness’ of the future self, a component of future self-identification, can be increased through exposure to the future self (McMichael et al., 2021). Thus, exposing people to their future self could increase their future self-identification.

**Step 3: Behavioral Change Techniques.**
Based on the identified mechanism in Step 2, we use the intervention technique ‘Exposure (to the future self)’ to trigger an increase in vividness of the future self (Marques et al., 2023; Michie et al., 2013).^a^

a For an elaborate description of the theoretical framework and how the BCTs map onto the theory, see Mertens et al., 2022a and Mertens et al., 2022b).

Alt-text: Unlabelled Box

## 3 DID-Guide: theory translation process

### 3.1 Step 4: overarching concept and narrative

Prior to turning to theory translation, it can be useful to contemplate an overarching concept for the intervention. This helps integrating the different components of the intervention's theoretical foundation (created in Steps 1–3). For instance, the Hospital Hero app (Poot et al., 2023), which aims to reduce preprocedural stress and anxiety for children visiting the hospital, uses a discovery map that guides children through their hospital visit as an overarching concept. This concept combines the components of the intervention's theoretical foundation, namely preparation, distraction, and support for caregivers (Poot et al., 2023). Another example is a VR intervention proposed by Slater et al. (2019) who used the overarching concept of counseling by a famous therapist, Sigmund Freud, to integrate the components of psychological distancing, perspective taking, and self-counseling. In the intervention, research participants alternated between virtually embodying an avatar representing themselves, and an avatar resembling Freud. While embodying their own avatar, they shared personal problems that they subsequently provided advice on, when embodying the avatar modeled on Freud.

An overarching concept lends itself to the creation of a narrative, which allows the technology to “tell a story” (see Case Example Step 4). A narrative allows characters and/or events to be embedded in a meaningful context and increases peoples' understanding of the design and purpose of the intervention (Reijers and Coeckelbergh, 2020). It creates a shared context in which Behavior Change Techniques (BCTs) can be operationalized in line with the narrative. This helps communicate the purpose of the intervention's assignments and interactions, which, in turn, may increase users' willingness to adhere to the intervention. Such a narrative can differ in the level of interaction with users. It can range from telling a story that users passively follow (e.g., peers that share personal experiences) to the creation of a digital world that users can engage with (Reijers and Coeckelbergh, 2020). Moreover, a narrative provides the opportunity to create an appealing storyline, and possibly also to incorporate (a certain level of) gamification, which makes it more attractive for the user to interact with the intervention (Kelders et al., 2018).

To illustrate, the biofeedback videogame MindLight (Schoneveld et al., 2016), which aims to reduce anxiety, uses a scary, dark mansion as an overarching concept. The narrative describes Arty, a character controlled by the user from a first-person perspective who needs to save his grandmother from evil forces in the haunted mansion. For this mission, Arty has a headlight that shines brighter when the user is calmer. In this example, the intervention tells a story using technology and creates an interactive context in which it makes sense for users to relax, as relaxation generates more light in the game. In other words, the BCT ‘Relaxation’ gets a purpose for users through the narrative of the intervention.

**Table:** 

**FutureU Case Example: Step 4.**

**Step 4: Overarching Concept and Narrative.**
The theoretical foundation of FutureU is based on future self-identification as an initiating and maintaining factor in delinquent behavior. The overarching concept revolves around technology-mediated interaction between a user and their future self. The narrative involves a time travel portal that allows for communication with the future self: it presents the future self as a person who the user can get to know better through interaction and by performing specific tasks and assignments.

Alt-text: Unlabelled Box

### 3.2 Step 5: choosing a delivery technology

Before deciding on a delivery technology, questions as to whether the use of technology has added value and whether there is an easier and/or less resource-intensive way to implement the intervention need to be addressed. When technology is considered to have merit, four interrelated aspects guide the choice for a delivery technology: 1) the target population and context, 2) ethical considerations, 3) the overarching intervention concept, and 4) the advantages provided by the technology (see Case Example Step 5).

#### 3.2.1 Target population and context

Target group members must both be motivated to use the technology and possess the skills and capacities to do so effectively (World Health Organization, 2019). For example, a smartphone-based intervention may be well-suited for an intervention that targets youth, but is perhaps not the ideal choice for the elderly for whom smartphone use is less likely to be integrated in their daily lives. In addition, the context in which people live, both on the macro-level, such as the country, and the micro-level, such as the family system, must enable technology use, for instance through accessibility of the delivery technology (World Health Organization, 2019).

#### 3.2.2 Ethical considerations

There are many ethical considerations related to the use of technology in digital interventions that are important to consider. One of these concerns data privacy. What data is (automatically) recorded and stored, who receives that data, and what can be done with it? For example, a VR headset could passively collect personal data about users, such as their body posture, pupil dilation, and what they are looking at (and at what not). A smartwatch can collect data about users' heartrate, geographical location, and number of steps. Although seemingly trivial, within a short time span numerous datapoints can be collected. When passed on to companies who developed the hardware, such as Meta or Microsoft, this provides an insight into their users. Such information can be used to improve the technology, but also for personalization of advertisements. Additionally, collecting this data increases the risk of identity hacking and blackmail (e.g., Slater et al., 2020; Wies et al., 2021).

Furthermore, technology provides increasing realistic experiences, especially applications that make use of Artificial Intelligence. The technology can give credible responses in reaction to the user, create personally relevant experiences, and simulate realistic scenarios. This enables deep manipulation of users' behaviors and cognitions without them being aware of it, threatening their autonomy. Moreover, it can fade the boundaries between reality and illusion paving the way for false hope and loosing oneself in this imaginative world (Madary and Metzinger, 2016; Slater et al., 2020; Wies et al., 2021).

Another important aspect, is the digital divide. Access to (more advanced) types of technologies is often limited in more deprived contexts (e.g., low income families; low income countries). Intervention developers should be cautious to further widening the digital divide and create a world in which interventions become less accessible for more vulnerable populations (Wies et al., 2021; World Health Organization, 2019).

Here we highlighted only three of many ethical considerations. As some ethical aspects are related to specific types of technology, we advise searching the literature for ethical considerations regarding the type of technology that are considered for use.

#### 3.2.3 Overarching intervention concept

A specific characteristic of digital interventions is that the technology must be able to express the overarching intervention concept. Relevant aspects to consider here are whether there is a need for visualization and what level of guidance is needed to convey the concept. If the concept is concrete and straightforward, for instance to increase users' daily number of steps, attractive design and data visualization may be sufficient, and users can interact with the intervention without additional guidance (e.g., Direito et al., 2018). With a more complex concept, such as simulating a hospital visit, visualization of the concept and step-by-step guidance may be necessary to bring it across (e.g., visualization of a hospital visit by means of animations; Poot et al., 2023).

#### 3.2.4 Technological advantages

Different technologies may offer overlapping possibilities, but may also have unique advantages. For example, both apps and VR offer possibilities for visualizing content, but differ in terms of how users can interact with the technology. Apps offer the opportunity for daily interaction in the natural environment, which may, for instance, better facilitate the formation of new habits. In contrast, VR offers the opportunity to immerse people in virtual environments, which may, for example, make it better positioned for users to acquire new skills. To illustrate, we briefly elaborate on several advantages of apps and VR below.

##### 3.2.4.1 Advantages of apps

People tend to carry their phone with them wherever they go. This allows users to interact with the intervention wherever and whenever they want, but also enables system-triggered intervention in real time within the ecological context users may need it most, i.e., ‘just-in-time’ interventions (Direito et al., 2018). For example, an app intervention targeting recovering alcoholics uses the smartphone's GPS to trigger automated intervention messages when users near a high-risk location, such as a bar (Gustafson et al., 2014). Intervention content can also be personalized based on data collected, for instance, by means of in-app questionnaires, sensor data, or app usage data. To illustrate, an app can measure user movement and subsequently adjust intervention goals for physical activity based on the tracked number of steps (Direito et al., 2018).

In short, apps are particularly useful for interventions that require frequent use, a low threshold for participation, in situ and real-time intervention, and addressing a heterogeneous population requiring personalized content. Furthermore, given that most people own a smartphone, there are no (hardware) costs for implementation.

##### 3.2.4.2 Advantages of VR

VR can psychologically transport users from their actual physical surroundings to virtual environments that are tailored to the purposes of the intervention, e.g., to practice or generalize certain skills. Users respond and behave realistically in virtual environments (Gonzalez-Franco and Lanier, 2017), showing to what extent users are able to generalize their newly acquired skills. To illustrate, Fromberger et al. (2018) used VR to perceptually transport sexual offenders to a supermarket where they encountered avatars they were attracted to. In this setting, they could practice their coping skills and therapists could observe their behavior.

Furthermore, in VR users can be represented by an avatar that, under the right conditions (e.g., synchronization of a user's own physical body movements with those of their avatar) can lead to the temporary illusion of embodiment (Bailey et al., 2016) or virtual body ownership. Under such conditions, the virtual body feels like one's own body even if the avatar's body looks different from the user's own physical appearance (Van Gelder, 2023). Embodiment facilitates perspective taking, which, in turn, can change attitudes and behaviors (Cornet and Van Gelder, 2020). For example, in a study by Seinfeld et al. (2018), male domestic violence offenders embodied a female avatar and experienced violent abuse from that perspective during a VR intervention. Following this experience their ability to recognize fear in faces was improved.

These characteristics make VR a suitable delivery technology for interventions focused on creating an immersive experience, acquiring and generalizing skills, experiencing embodiment, and facilitating perspective taking.

### 3.3 Step 6: usage aims

After choosing the technology for intervention delivery, usage aims can be formulated. Usage aims address (maintaining) engagement with the intervention in general and/or with specific intervention components (Mohr et al., 2014), i.e., how the technology should be used to achieve the intervention aims. As such, usage aims may concern the use of tools representing BCTs, but could also focus on engagement with the intervention or ease of use (Mohr et al., 2014). Examples of usage aims are: Interaction with intervention tools, independent use of the intervention application, and (frequent) exposure to the intervention's content. In addition, usage aims should align with typical use and the advantages of the delivery technology, e.g., the usage aim ‘daily use of the intervention tools’ could work well for an app intervention but is less suitable for a VR intervention (see Case Example Step 6).

**Table:** 

**FutureU Case Example: Steps 5 and 6.**

**Step 5: Delivery technology.**
FutureU aims to decrease self-defeating behavior by increasing future self-identification. However, imagining the future self can be difficult. Presenting an image of this future self could reduce both the cognitive burden and the reliance on people's imaginative abilities. Hence, technology can be of added value to the intervention as it enables a visual rendering of the future self.

For the overarching concept of FutureU, technology needs to enable visualization of the future self and allow for interaction with an avatar representing it. Both apps and immersive VR allow for this. Additionally, both technologies appeal to the target population, young adults, which could increase their motivation to participate and stimulate treatment adherence.

The initial target population of FutureU were offenders, both in the community and in prisons. A smartphone-based intervention was considered a better fit for the group living in the community, as they could use it in their own environment at any time, whereas a VR intervention was considered more suitable for use inside prisons, as inmates remain in the same location and are not allowed to have smartphones. Therefore, FutureU was developed for both types of technology.

**Step 6: Usage aims.**
App: Given that apps are optimally suited for frequent interaction, we aimed at developing features within the app that are user-friendly and which are used multiple times.
Usage aim of the FutureU app: Stimulating frequent exposure to, and interaction with, the future self, and use of the features within the app.

VR: Given that VR is optimally suited for creating immersive experiences allowing for virtual embodiment, we opted for an experience in which users embody avatars representing their present and their future selves.
Usage aims of FutureU VR: Generating an immersive and embodied experience in which users ‘become’ their future self.

Alt-text: Unlabelled Box

### 3.4 Step 7: conceptualization of Behavior Change Techniques (BCTs)

In this step, the BCTs identified in Step 3 are converted into conceptual ideas that align with the overarching intervention concept created in Step 4. In other words, how can the intervention techniques be operationalized in line with the overarching concept of the intervention so that the interactions and assignments make sense to users? In the biofeedback videogame MindLight, for instance, the overarching concept, is a dark, haunted mansion, and the BCT ‘Relaxation’ is operationalized by bringing light into this darkness (Schoneveld et al., 2016). As it is difficult to see in the dark, it intuitively makes sense to have an exercise to create light. Hence, developing concepts for BTCs can be regarded as developing assignments and exercises that fit within the overarching concept in a meaningful way (see Case Example Step 7).

When brainstorming about conceptual ideas for a BCT, it can help to define the exact aim of that BCT and to create a short narrative within the overarching concept. To illustrate, the aim of the BCT ‘Relaxation’ is to make people calmer. In a dark, haunted mansion, people will typically become calmer once they can see more. So, the narrative of MindLight around ‘Relaxation’ revolves around bringing light into the darkness.

**Table:** 

**FutureU Case Example: Step 7.**

**Step 7: Conceptualization of BCTs.**
The overarching intervention concept of FutureU is technology-mediated interaction between users and their future selves. Inline with this, we gave the future self a central role for each BCT and translated the concept into different assignments and exercises. For example, the BCT ‘Exposure (to the future self)’ aims to expose users to a rendering of their future self. To make sense of seeing your future self, we created the narrative in which users need to establish a connection with their future self to enable interaction. The BCT ‘Perspective taking’ is aimed at psychological distancing and perspective taking on problems. To this end, we developed a narrative in which users travel through time and become their future self in order to give their younger, i.e., current, self-advice. The BCT ‘Episodic Future Thinking’ aims to foster planning for the future by ‘pre-living’ future events through imagination. Therefore, we created the narrative in which users describe a specific event their future self has experienced.

Alt-text: Unlabelled Box

### 3.5 Step 8: converting conceptual ideas into features

**Fig. 3:**
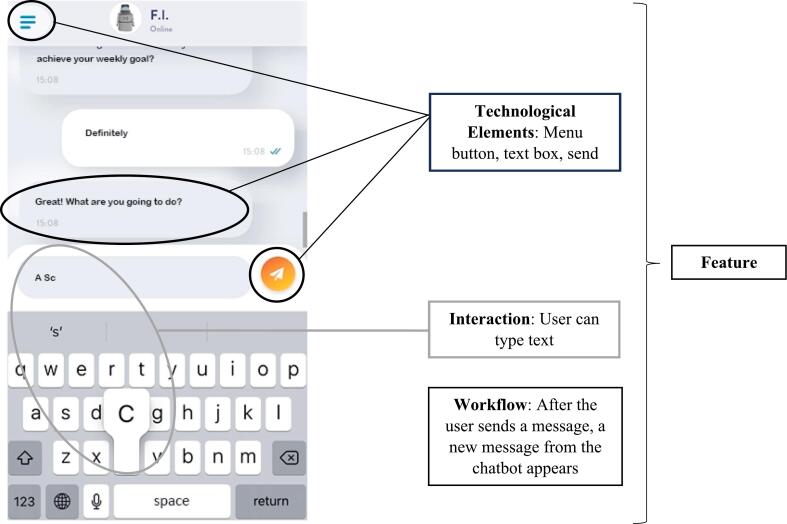
Screenshot of the Chat-feature of the FutureU App. It Shows the Different Development Steps Needed to Build a Feature: Technological Elements, Interactions, and Workflows.

In this step, the concepts of the BCTs are translated into concrete features within the digital intervention. A feature is composed of *technological elements* that the user can *interact with* according to a predefined *workflow*. For example, a chat-feature roughly consists of technological elements that can show the received and sent messages, and an element (e.g., a keyboard) that enables users to send messages. Users can interact with these elements by reading the messages and sending new ones. The workflow has a specific order: 1) users type a message, 2) send it, and 3) the message pops up in the chat (see Fig. 3).

In line with the BIT framework (Mohr et al., 2014), the conversion process from BCT concepts into features consists of three stages: 1) determining each feature's required technological elements, 2) defining and refining user interactions, and 3) programing workflows. In other words: *What* is needed, *how* can it be used, and *when* is it presented? These three steps are intertwined in the sense that changes in one step often require adjustments in the other steps. For instance, if the chat-feature is adjusted so that users cannot send messages anymore, both the technological elements and the workflow need to be adjusted accordingly. Hence, in the development process of features, it is typical to go back and forth between the steps, rather than treating them sequentially.

Below, we will further elaborate on the three stages, i.e., the What, the How, and the When, including a Case Example of an app feature (see Case Example Step 8 – App) and a VR feature (see Case Example Step 8 – VR). Additionally, as currently many DMH interventions are developed for apps and VR, we provide additional details on feature development for these two technologies in the Appendix.

#### 3.5.1 Step 8a: the “what”

The technological elements required to build a specific feature have to be identified. What needs to be presented to users to enable them to understand and use the feature correctly? There are different types of technological elements, such as elements for information delivery, notifications, data entry, passive data collection, and visualization, all of which can be combined. To illustrate, a goal-setting feature to increase physical activity can have technological elements, such as a menu to present and visualize the goal, passive tracking of steps, automatized calculation of a goal based on the tracked steps, and automated presentation of the personalized goal in the menu (e.g., Direito et al., 2018). To give another example, the chat-feature in Fig. 3 requires a chat interface, messages that are sent and received, a keyboard for typing messages, and a menu button.

#### 3.5.2 Step 8b: the “how”

Technological elements should be presented in such a way that users understand what is expected from them and want to engage with the feature. To illustrate, the feature presented in Fig. 3 resembles a typical chat interface in terms of layout and functionality. As a result, users intuitively understand how they can use it to send and receive text messages.

In this step the ‘digital experience of the user’ is designed, which consists of the user experience (UX) and the user interface (UI). UX concerns the design of functional and logical interactions features as well as the complete intervention. UI involves the look, appeal and aesthetics of the specific elements, features and complete intervention. The UX and UI design support the usability, usefulness, and desirability of interactions with features, resulting in user-friendly interactions (Stige et al., 2023). UX and UI design can optimize the fit with a feature's purpose (such as providing information) and/or target population (such as youth or the elderly) and can make an interaction either easier or more challenging. This can be established through, for example, the type of media used (e.g., text, video, audio), the complexity of interactions (e.g., multiple choice versus free text entry), aesthetics, and personalization. To illustrate, in the MyFitnessPal app (Mohr et al., 2014), a food monitor feature has a technological element to enter food intake using free text input. To accommodate the user, this element is refined with additional interaction options: 1) selecting a particular food from a predefined list, and 2) scanning the barcode on the packaging of the food item.

#### 3.5.3 Step 8c: the “when”

When and under what circumstances an element is delivered, i.e., the workflow of the technological elements, needs to be decided when developing a feature. In Fig. 3, the workflow mirrors a typical chat interaction. First, the chatbot sends a message. Then there are two options: 1) the chatbot sends a new message after a few seconds (to give users time to read the first message before providing the new information) or 2) users respond to the chatbot's message. When users respond, the workflow is as follows: a) the user types a message that appears in the textbox, b) the user presses the send button, c) the user's message appears in the chat interface, and - optionally - d) the user's message triggers a new message from the chatbot.

There are different types of workflows that can be used to deliver technological elements, for example instant delivery, delivery with a certain frequency, delivery after a specified time period or event, delivery upon completion of a task, or delivery based on collected data (personalization). To illustrate personalized delivery, in the app by Direito et al. (2018), users receive a push notification when their moving behavior falls below their weekly goal for five consecutive days. Delivery after an event is illustrated in the app by Fanning et al. (2017), where a new feature becomes available after watching a short video. Similarly, in the VR game Walls (Farič et al., 2021), users receive a more challenging task each time they complete one.

**Table:** 

**FutureU Case Example: Step 8 – Apps.**

**Step 8: Converting Conceptual Ideas into Features for the FutureU App.**

**The App Feature.**
For the BCT ‘Exposure (to the future self)’, we developed a ‘connection mechanic’ that presented users with an avatar resembling their future self.

**Fig. 4:**
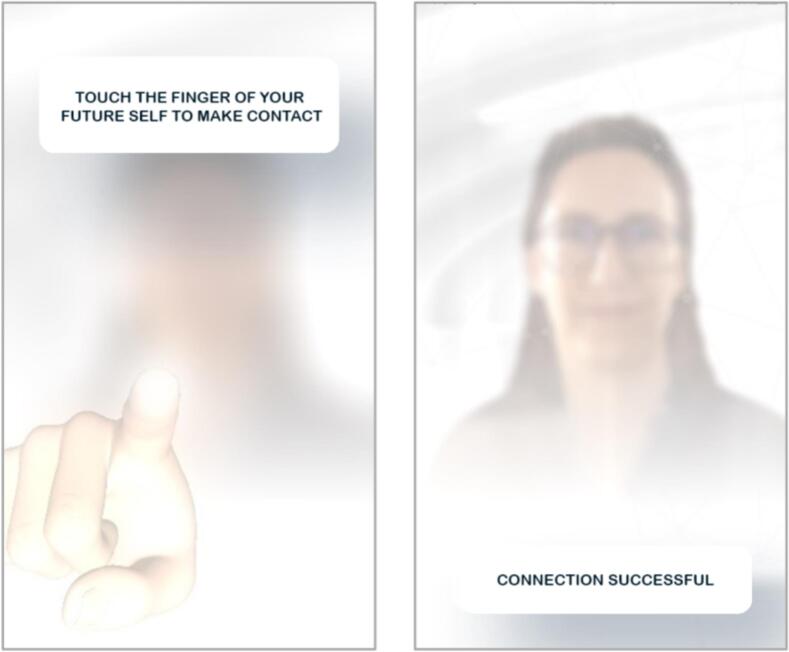
Screenshot of the FutureU App Showing the Connection Mechanic Feature. This Feature is Developed to Operationalize the Behavior Change Technique ‘Exposure’: During this Feature Users are Exposed to a Visual Image of Their Future Self.

**Overview Connection Mechanic.**
Users receive a message from their future self at a specific time (push notification). Clicking on the notification opens a screen showing a blurred headshot of their future self. The narrative accompanying the interaction is that users need to make contact with their future self to enable a conversation with it. Contact is established by touching the virtual finger of their future self on the screen for a few seconds. Doing this unblurs the future self and enables the user to progress to the next feature (Fig. 4).

**Step 8a: Technological Elements** - Push notification - Button on the home screen - Text boxes with instructions (e.g., “Touch the finger of your future self”) - Avatar of the future self (based on a ‘selfie’ taken by the user) - Filter to age the selfie - Filter to blur and unblur the future self-avatar - Vibration of the phone when users touch the virtual finger - Audio cue when the avatar unblurs

**Step 8b: Interactions.**
The interaction with the avatar of the future self is straightforward and involves touching the virtual finger of the future self for several second. This active involvement of users ensures that they are paying attention to the app. During the interaction, the phone vibrates and plays a sound as an additional attention grabber.

**Step 8c: Workflows.**
The connection mechanic feature becomes available on time-based rules: The workflow is preprogrammed to enable its use only on specified days. Within the feature, some elements appear in combination, such as the instruction text boxes, the avatar, and the blurring filter. The workflow of the (combined) elements is mainly task-based, i.e., after clicking the notification, the connection mechanic opens and after touching the virtual finger for 4 s, the future self unblurs.

Alt-text: Unlabelled Box

**Table:** 

**FutureU Case Example: Step 8 – VR.**

**Step 8: Converting Conceptual Ideas into Features for the FutureU VR.**

**The VR Feature.**
The BCT ‘Exposure (to the future self)’ was operationalized as a digital avatar of the future self that users can see as well as embody.

**Fig. 5:**
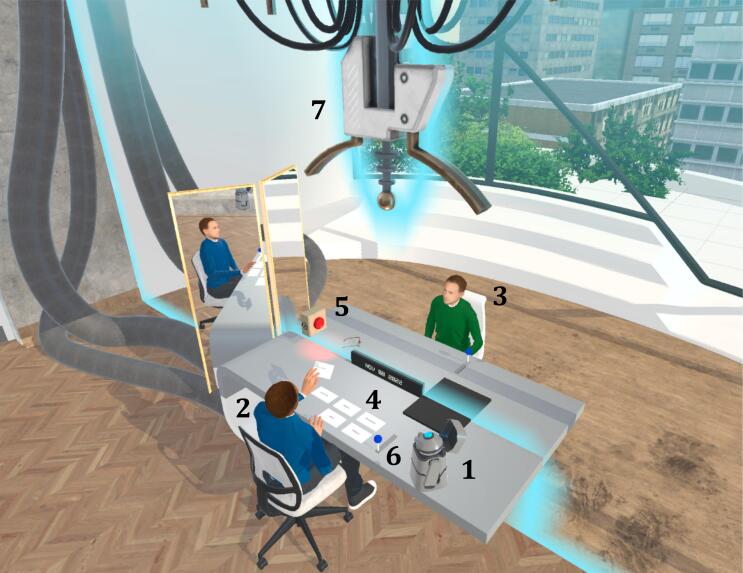
Overview of the Virtual Environment of FutureU with the Different Technological Elements. *Note*. 1 = Robot controlled by researcher; 2 = Present self-avatar; 3 = Future self-avatar; 4 = Cards with questions; 5 = Recording light; 6 = Lever to activate time machine; 7 = Time machine.

**Overview Exposure to Future Self-Avatar.**
In FutureU VR there are two avatars: One representing the present self and the other representing the future self. These two avatars sit on opposite sides of a table facing each other (Fig. 5). The narrative explains that users are in a time portal that enables them to have a conversation with their future self and travel through time. Users are exposed to their future self when embodying their present self-avatar (and face their future self-avatar sitting opposite of them) and when embodying their future self-avatar (and looking into the mirror).

**Step 8a: Technological Elements** - Table - Chairs - Time machine - Lever to activate time machine - Visual supporting ‘time travel’ - Audio supporting ‘time travel’ - Windows including a view on contemporary urban landscape buildings and a futuristic landscape - Mirrors - Hoovering robot controlled by the researcher (or experimenter) - Full-body avatar of the present self (based on a ‘selfie’ taken by the user) - Full-body avatar of the future self (based on a ‘selfie’ taken by the user) - A filter to age the selfie for the future self-avatar - Avatar creation tool - Shadows of the avatars and objects

**Step 8b: Interactions.**
As future self, users look in the mirror and describe aloud what has changed about their appearance compared to the present self-avatar. Additionally, embodiment of the future self-avatar is strengthened with a scripted embodiment exercise in front of the mirror during which users experience that their real-world movements (e.g., raising their hand) are synchronized with the virtual movements of the avatar.

**Step 8c: Workflows.**
The workflow is determined by the script of the VR session that the experimenter follows.

Alt-text: Unlabelled Box

#### 3.5.4 Mapping BCTs on features

During the conversion process, it can become apparent that multiple BCTs map onto the same or related concepts and can thus be operationalized in the same feature (e.g., expose users to their future self [BCT: Exposure] while they also take on the perspective of this self [BCT: Perspective taking]). Conversely, it can also be that one BCT maps onto multiple concepts and, thus, can be operationalized in multiple features (e.g., use the visualization of the future self [BCT: Exposure] in multiple features).

Mapping the concepts of the BCTs on specific features results in ideas for features with clear underlying concepts and BCTs. This facilitates communication about these ideas to others, such as designers and software developers. Clear communication about the ideas for features is particularly important when working with third parties that develop the technological aspects in order to ensure the accurate translation of the theoretical BCTs into technological elements, interactions and workflows.

## 4 Conclusion

The future of mental health interventions using technology is promising, as technology creates novel opportunities. For example, apps allow for large-scale implementation – reaching a broader and more diverse population – and yield many opportunities for (automated) personalization. VR can transport people to interactive and immersive virtual environments that can be tailored to meet individual needs. Recent developments in generative Artificial Intelligence can further enhance the potential of Digital Mental Health (DMH) interventions, for example, through the deployment of intelligent chatbots and automated supervision of interventions. Regardless of technological development, the process of translating theory into features will remain an important step in DMH intervention development.

The DID-Guide can provide a comprehensive resource for anyone (e.g., researchers, clinicians, developers) who intends to develop a DMH intervention from theory building to actual technological features of the intervention. The two-phase structure of the guide enables flexible application: It can be used as a stand-alone guide to develop a DMH intervention from scratch by following the steps of both phases, or it can be used for (additional) guidance in the theory translation process by following the steps of phase two, for instance, when the intervention's theoretical foundation is already formulated (e.g., when converting an existing in-person intervention into a digital intervention). Moreover, following the steps of the DID-Guide ensures a clear rationale for each feature of the digital intervention which facilitates effective communication and collaboration between different stakeholders. In sum, the DID-Guide aims to support both the development of DMH interventions and communication between the involved parties.

## Funding sources

The study is financially supported by the ERC Consolidator Grant (Grant Number 772911-CRIMETIME). The funder has no role in the study design, collection, analysis and interpretation of data, and in the writing of manuscripts.

## Declaration of competing interest

The authors declare the following financial interests/personal relationships which may be considered as potential competing interests: Jean-Louis van Gelder reports financial support was provided by European Research Council. If there are other authors, they declare that they have no known competing financial interests or personal relationships that could have appeared to influence the work reported in this paper.

## Appendix A

### Features for apps and VR

This section discusses the development of features for apps and VR by describing possible technological elements, interactions, and workflows for these two technologies.

### A.1 Apps: possible technological elements, interactions, and workflows

#### A.1.1 Technological elements

Apps allow for frequent use, automated delivery of intervention content, and passive data collection (Linardon et al., 2019). Multiple technological elements align with these advantages. For example, push notifications can remind users to interact with a specific feature in the app or provide intervention content. Interactive experiences can be facilitated by technological elements that require user input, such as text boxes for users to enter data (Mohr et al., 2014), the possibility to scan QR codes via the smartphone's camera to access intervention related content (Poot et al., 2023); or a menu with the option to send messages to a specific recipient, for instance a therapist (Gustafson et al., 2014). Another advantage specific to apps is that they can use one or more of the many sensors integrated in smartphones. To illustrate, technological elements can use the smartphone's built-in accelerometer to log the movement of users for a physical activity intervention (Direito et al., 2018), use the smartphone's GPS to alert users when they are near a bar in an intervention for recovering alcoholics (Gustafson et al., 2014), or use the smartphone's camera to scan a QR code (Poot et al., 2023).

#### A.1.2 Interactions

UX and UI design should be based on general patterns of app use. That is, users typically interact with an app for brief moments at a time and do so multiple times a day (Morrison et al., 2018). Generally, apps are used without (therapist) supervision, which provides flexibility in timing of delivery of intervention content but also places limits on the types and amount of explanation that can be provided. It follows that interactions are ideally brief and self-explanatory, and the option of stopping and starting again at any given time in the process allows for flexibility.

User-friendly interactions are characterized by UX and UI design that facilitate quick and easy navigation. This can entail an overview of the features including buttons for direct access to these features or restricting the number of available features to limit the choice-options in the app (Morrison et al., 2018). In conjunction with user-friendly navigation, different types of media (e.g., text, videos, graphs) can support interactions. To illustrate, information can be provided visually by means of animation instead of a lengthy text or interactively by means of messages sent by a chatbot to which users respond.

#### A.1.3 Workflows

Apps allow for a broad range of typically preprogrammed workflows, such as time-based rules, task-completion rules, and event-based rules (Mohr et al., 2014). These rules can also be combined. To illustrate, in the app by Direito et al. (2018) the goal feature automatically sets a goal for the user each day (time-based rule). If the user reaches the goal, a smiley face is presented (task-completion rule). If the user does not meet the goal for five consecutive days, a push notification is sent (event-based rule).

Besides predefining the workflow for technological elements within a feature, a workflow can also be defined between features within an app to determine when and under which circumstances the different features are available. To illustrate, all features can be available at all times (no rule), features can become available over time (time-based rule) or upon completion of another feature (task-completion rule), or features can be prohibited from recurrent use (event-based rule).

### A.2 VR: possible technological elements, interactions and workflows

#### A.2.1 Technological elements

VR allows for immersive experiences where the illusion of being mentally present in a virtual environment is created. This illusion can be strengthened by matching real-world sensory input with virtual input (Cornet and Van Gelder, 2020) and/or synchronizing movements of the user's body in the real-world with the movements of the avatar in the virtual environment. For example, if users are sitting on a chair in the real-world, they also sit on a chair in the virtual environment. When users raise their arm in the real-world, their avatar also raises its arm (e.g., Farič et al., 2021).

#### A.2.2 Interactions

Typically, VR is interactive and follows a narrative to engage users cognitively and emotionally with the virtual environment (Cornet and Van Gelder, 2020). To make the experience interactive, technological elements that users can manipulate can be used (e.g., picking up an object, pushing a button, opening a door) so that they can influence the way the scenario unfolds. Interactions can also be facilitated through different types of media, such as conversations, texts, and videoclips. For example, in a growth mindset VR intervention (Schleider et al., 2019), users can select which scenes to participate in, can talk aloud to other avatars, and receive information both verbally and through animations.

VR interventions often occur under supervision of a facilitator who can guide or influence the interaction. This allows for more challenging interactions, assignments, and personalization, as the facilitator can provide support and personalize content. For instance, in the YourSkills VR intervention, which aims to reduce aggression in children (Alsem et al., 2023), facilitators can manipulate the virtual situation, such as turning off the virtual television or letting the user lose a game, as well as control the actions and speech of agents in the environment. Furthermore, aesthetics can be used to increase users' acceptance of the virtual environment (Mohr et al., 2014). Note, however, that in VR, well-designed aesthetics are not necessarily naturalistic representations of people and objects. In fact, realistic aesthetics can even be counterproductive by evoking unsettling feelings in users if digital humans appear almost lifelike, but just fall short thereby generating an eerie feeling, an effect known as the ‘uncanny valley’.

#### A.2.3 Workflows

The workflow in VR interventions is often determined by the facilitator guiding the VR experience. The facilitator can, for example, follow a protocol or script, select which scenario to play, and (de)activate features. Alternatively, the workflow can (partly) be determined by the user, by manipulating technological elements in the virtual environment or making certain choices they are confronted with in the virtual environment, which, in turn, activates other features. To illustrate, in the VR intervention of Schleider et al. (2019), users can choose which assignment they do and which agents they speak to.
